# Impact of pathologically diagnosed metabolic dysfunction-associated steatohepatitis on weight loss outcomes following sleeve gastrectomy

**DOI:** 10.3389/fmed.2026.1780788

**Published:** 2026-03-10

**Authors:** Yunmiao Pan, Yunfei Qu, Hanchen Ma, Maoge Wang, Mingwei Zhong, Sanyuan Hu

**Affiliations:** 1 Department of General Surgery, Shandong Provincial Qianfoshan Hospital, Cheeloo College of Medicine, Shandong University, Jinan, China; 2 Department of General Surgery, Peking University People's Hospital Qingdao Hospital, Qingdao, China; 3 Department of General Surgery, The First Affiliated Hospital of Shandong First Medical University and Shandong Provincial Qianfoshan Hospital, Jinan, China; 4 Laboratory of Metabolism and Gastrointestinal Tumor, The First Affiliated Hospital of Shandong First Medical University and Shandong Provincial Qianfoshan Hospital, Jinan, China; 5 Department of General Surgery, Qilu Hospital, Cheeloo College of Medicine, Shandong University, Jinan, China

**Keywords:** bariatric surgery, laparoscopic sleeve gastrectomy, metabolic dysfunction-associated steatohepatitis, obesity, weight loss

## Abstract

**Purpose:**

The impact of metabolic dysfunction-associated steatohepatitis (MASH) on post-operative weight loss outcomes remains unclear. This study aims to investigate the effects of preoperative concomitant MASH on weight loss outcomes and metabolic improvements following laparoscopic sleeve gastrectomy (LSG).

**Methods:**

A retrospective analysis was performed on the clinical data of 226 patients with obesity who underwent LSG and concurrent intraoperative liver biopsy. Univariate analysis, multivariate analysis, and general linear models were employed to evaluate differences in the dynamic trajectories of post-operative weight loss between the groups. Additionally, Kaplan–Meier survival analysis was utilized to compare the time required to achieve successful weight loss outcomes.

**Results:**

Preoperative body mass index (BMI) and the presence of MASH were independent negative predictors of percentage excess weight loss (%EWL) and percentage total weight loss (%TWL) (*P* < 0.01). The post-operative %EWL difference between the two groups reached up to 28.9%. Compared with non-MASH patients, patients with preoperative MASH had a significantly lower cumulative incidence of achieving 80% EWL within 1 year post-operatively (χ^2^ = 35.17, *P* < 0.05, HR = 2.058).

**Conclusion:**

Preoperative MASH is significantly associated with lower post-operative weight loss. Compared to non-MASH patients, patients with MASH experienced a delay in achieving satisfactory weight loss outcomes within the first year after surgery, and this was significantly associated with a lower post-operative %EWL.

## 1 Introduction

In recent years, obesity has emerged as one of the most serious metabolic diseases worldwide (1). Studies have shown a significant increase in the prevalence of obesity in China, with a notable trend toward younger age groups (2). Notably, obesity is not merely a matter of excess weight; it is associated with a spectrum of metabolic disturbances and serves as a pivotal initiating factor for metabolic syndrome. Against this backdrop, metabolic dysfunction-associated steatotic liver disease (MASLD) has developed into the leading chronic liver disease globally. During the progression of this disease, metabolic dysfunction-associated steatohepatitis (MASH) is often regarded as a critical and severe pathological stage. The prevalence of MASLD is rapidly increasing globally, particularly among individuals with obesity and diabetes (3). MASLD is present in approximately 90% of morbidly obese individuals, with progression to MASH occurring in up to 65% of cases (4). The core mechanism involves a vicious cycle of insulin resistance, chronic inflammation, and dysregulated lipid metabolism, which drives hepatic inflammation and fibrosis while significantly increasing the risks of cardiovascular disease and type 2 diabetes mellitus (T2DM) (5–7).

As chronic progressive diseases, obesity and MASLD follow a long-term course that not only leads to liver-related complications, such as cirrhosis and hepatocellular carcinoma (HCC), but is also closely linked to systemic inflammation and an elevated risk of cardiovascular events, thereby significantly impairing patients' quality of life (8). Furthermore, research indicates that obese patients with MASH frequently experience fatigue, abdominal discomfort, and an increased psychological burden (9). Compared to those with simple obesity, patients with MASH exhibit a higher prevalence of psychiatric comorbidities, including psychological trauma, depression, and social impairment (10). Notably, evidence suggests that up to 25% of MASH patients suffer from depression and social withdrawal (11). Given the chronic nature of these conditions, the implementation of long-term management strategies is essential to alleviate the profound burden they impose on both individuals and society.

Lifestyle modification aimed at weight reduction remains the cornerstone of MASH management (12). Among therapeutic interventions, bariatric surgery, such as laparoscopic sleeve gastrectomy (LSG), has emerged as a highly effective approach. LSG is increasingly favored for its favorable safety profile, low complication rate, and proven efficacy in inducing sustained weight loss. Furthermore, accumulating evidence demonstrates the benefits of LSG in promoting MASH resolution and improving hepatic histology (13–16).

However, while existing studies have largely focused on the histological benefits of LSG, few have investigated whether preoperative MASH status independently influences post-operative weight loss trajectories and metabolic outcomes. This paucity of data limits clinical decision-making, specifically regarding whether differentiated perioperative management strategies are required for obese patients with concomitant MASH. To address this, our study utilized intraoperative liver biopsies to precisely stratify patients into MASH and non-MASH groups. By systematically analyzing weight loss trends and metabolic variations over a 1-year post-operative period, we aimed to determine whether MASH affects the weight-loss efficiency of LSG independently of potential confounders, while also exploring whether patients with varying hepatic pathological stages exhibit time-dependent patterns in metabolic improvement. Our findings aim to provide a novel evidence-based foundation for the personalized surgical assessment of patients with obesity and MASH.

## 2 Materials and methods

### 2.1 Participants

**Figure 1:**
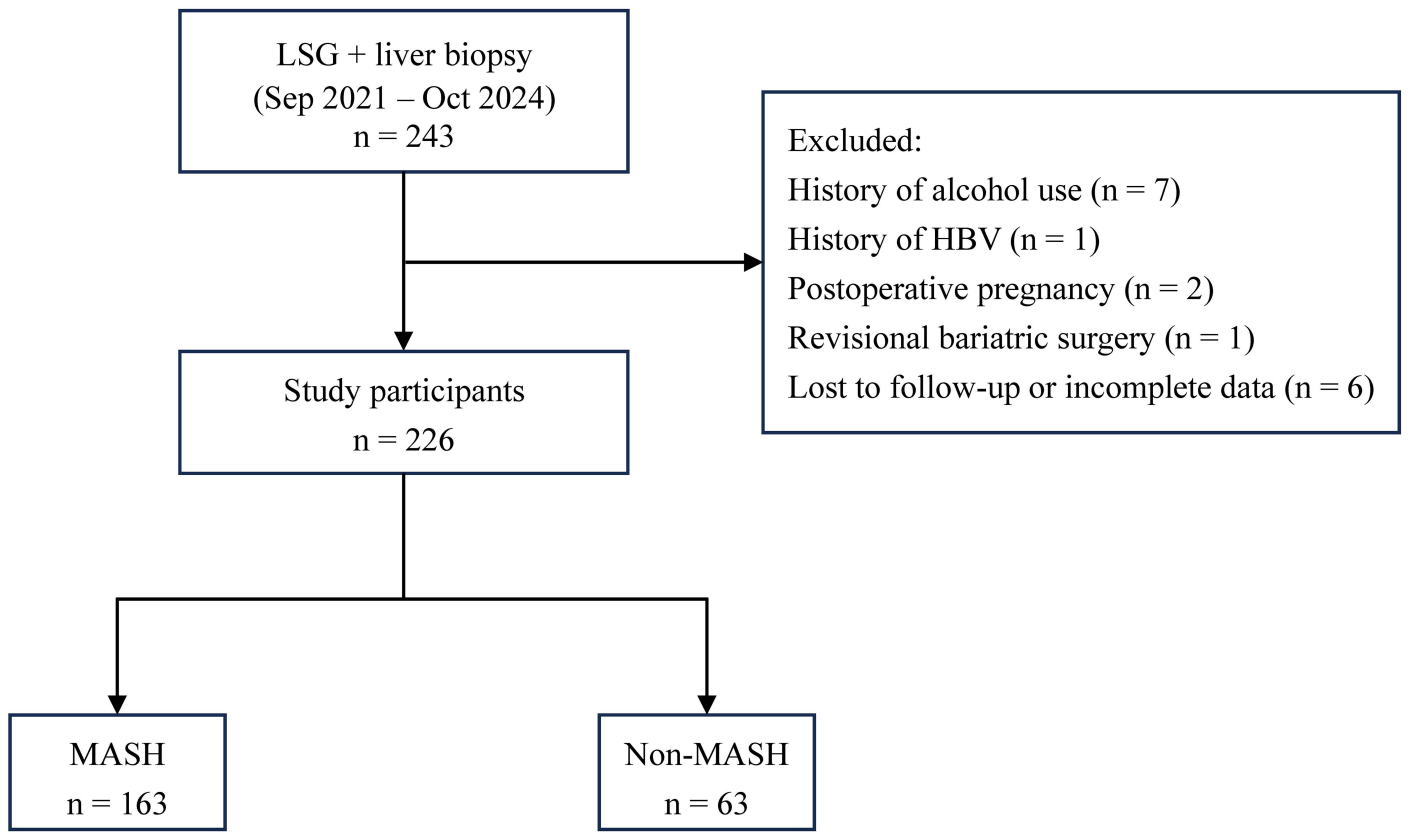
Flowchart of study population selection and categorization. LSG, laparoscopic sleeve gastrectomy; HBV, Hepatitis B; MASH, metabolic dysfunction-associated steatohepatitis.

This retrospective study enrolled a total of 226 patients with obesity who underwent primary LSG with concurrent intraoperative liver biopsy at Shandong Provincial Qianfoshan Hospital between September 2021 and October 2024. Given that LSG accounts for the vast majority of bariatric procedures at our center, only patients undergoing primary LSG were included to ensure a homogeneous study population. The criteria for inclusion were established as follows: (1) age between 16 and 65 years; (2) body mass index (BMI) ≥ 32.5 kg/m^2^; (3) BMI between 27.5 kg/m^2^ and 32.5 kg/m^2^ with the presence of obesity-related comorbidities; (4) completion of intraoperative liver biopsy; and (5) availability of complete post-operative follow-up data. The exclusion criteria included: (1) a prior history of bariatric surgery; (2) significant alcohol consumption; (3) use of hepatotoxic medications; (4) a history of preexisting liver diseases; (5) incomplete follow-up data; (6) patients with poor compliance or those who utilized weight-loss medications (e.g., GLP-1 receptor agonists) post-operatively were excluded to minimize confounding; and (7) patients who required reoperation or revision surgery during the 12-month follow-up period. This investigation was approved by the Institutional Ethics Committee of Shandong Provincial Qianfoshan Hospital, No. 2025(S035). The patient selection process is illustrated in Figure 1.

### 2.2 Clinical and laboratory data

Demographic and clinical data were extracted from the electronic medical record system, including gender, age, height, weight, BMI, waist-to-hip ratio (WHR), history of alcohol consumption, and obesity-related comorbidities such as hypertension, dyslipidemia, impaired glucose tolerance, and T2DM. Laboratory parameters included aspartate aminotransferase (AST)/alanine aminotransferase (ALT), uric acid (UA), triglycerides (TG), total cholesterol (TC), high-density lipoprotein cholesterol (HDL-C), low-density lipoprotein cholesterol (LDL-C), free fatty acids (FFA), glycated hemoglobin (HbA1c), and the homeostatic model assessment of insulin resistance (HOMA-IR) (17). Liver tissue processing and pathological diagnosis were performed independently by two pathologists under blinded conditions (18, 19). All patients received standardized preoperative education and post-operative instructions, which included structured counseling on diet and medication provided by trained clinical staff. Post-operative follow-up visits were conducted at 1, 3, 6, and 12 months. These assessments included post-operative weight measurement, blood tests, evaluation of surgery-related complications, and monitoring of improvements in obesity-related comorbidities. The percentage of excess weight loss (%EWL) was used as the primary metric to evaluate the efficacy of the bariatric surgery. The ideal body mass index (IBMI) was defined as 25 kg/m^2^ (20).


$$\begin{array}{l} \;\%TWL=(initial\;body\;weight\;-\;final\;body\;weight)/\\ \;(initial\;weight)\;\times \;100\%\\ \;\%EWL=([initial\;body\;weight\;-\;final\;body\;weight]/\\ \;[initial\;weight\;-\;ideal\;body\;weight])\times 100\%\\ HOMA-IR\;=(Fasting\;Glucose\;\times Fasting\;Insulin)/\text{(22.5)} \end{array}$$

### 2.3 Statistical analysis

The normality of data distribution was assessed using the Kolmogorov-Smirnov test. Normally distributed data were expressed as mean ± standard deviation (SD), while non-normally distributed data were presented as median and interquartile range. Differences between groups were compared using the Chi-square test, independent samples *t*-test, or Kruskal–Wallis test, as appropriate. Multivariable linear regression analysis was performed to investigate the independent impact of MASH on post-operative outcomes. A general linear model was utilized to analyze the dynamic changes in %EWL between the two groups. Additionally, Kaplan–Meier survival analysis was employed to compare the time required to achieve a favorable weight loss outcome (defined as ≥80% EWL) between the groups. Data analysis and graphing were performed using IBM SPSS Statistics 27.0 and GraphPad Prism 9.5.0 software, respectively. A two-sided *P*-value < 0.05 was considered statistically significant.

## 3 Results

### 3.1 Baseline characteristics of the study population

A total of 226 patients undergoing LSG were included in this study, of whom 68.58% were female. The median age of the entire cohort was 29 (25–35) years. Overall, the median weight, BMI, and WHR were 115 (103–134) kg, 40.52 (36.49–45.70) kg/m^2^, and 0.96 (0.90–1.00), respectively. Regarding obesity-related comorbidities, the prevalence rates were 40.27% for T2DM, 34.51% for HTN, 57.07% for obstructive sleep apnea (OSA), and 37.61% for metabolic syndrome (MetS).

Based on histopathological evaluation, patients were stratified into the non-MASH group (n = 63) and the MASH group (*n* = 163). The baseline clinical and biochemical characteristics of the two groups are summarized in Table 1. No significant differences were observed between the two groups regarding age, gender distribution, prevalence of HTN, OSA, MetS, FFA, TC, HDL-C, or LDL-C. However, several metabolic and anthropometric parameters differed significantly. Patients with MASH exhibited higher WHR (0.97 ± 0.07 vs. 0.92 ± 0.07 *P* < 0.001) and BMI [42.10 [37.89–47.12] vs. 37.74 [34.80–40.88] kg/m^2^, *P* < 0.001]. They also had significantly elevated levels of UA (*P* = 0.002), HbA1c (*P* = 0.010), TG (*P* = 0.008) and HOMA-IR (*P* < 0.001). Conversely, the AST/ALT ratio was significantly lower in the MASH group (*P* < 0.001).

**Table 1:** Baseline characteristics of patients with and without MASH.

**Variable**	**Non-MASH (*n* = 63)**	**MASH (*n* = 163)**	***r*/*X*^2^**	***P***
Age (years)	31 (25–38)	30 (25–35)	−0.053	0.428
Gender *n* (female %)	49 (77.8%)	106 (65.0%)	3.427	0.064
WHR	0.92 ± 0.07	0.97 ± 0.07	0.250^***^	< 0.001
BMI (kg/m^2^)	37.74 (34.80–40.88)	42.10 (37.89–47.12)	0.348^***^	< 0.001
T2DM *n* (%)	19 (30.2%)	72 (44.2%)	3.710	0.054
HTN *n* (%)	19 (30.2%)	59 (36.2%)	0.733	0.392
OSA *n* (%)	33 (52.4%)	96 (58.9%)	0.787	0.375
MetS *n* (%)	18 (28.6%)	67 (41.1%)	3.042	0.081
AST/ALT	0.79 (0.62–0.86)	0.65 (0.54–0.78)	−0.289^***^	< 0.001
UA (umol/L)	370.29 ± 92.32	416.85 ± 101.19	0.201^**^	0.002
HbA1c (%)	5.8 (5.5–6.2)	5.9 (5.6–6.9)	0.172^*^	0.010
HOMA-IR	6.17 (4.17–9.55)	9.01 (5.94–14.89)	0.275^***^	< 0.001
FFA (mmol/L)	0.52 (0.40–0.75)	0.62 (0.49–0.76)	0.132	0.056
TG (mmol/L)	1.23 (1.01–1.86)	1.58 (1.22–2.40)	0.177^**^	0.008
TC (mmol/L)	4.35 (3.74–5.04)	4.59 (4.03–5.10)	0.122	0.069
HDL-C (mmol/L)	1.06 (0.94–1.17)	1.03 (0.90–1.20)	−0.025	0.712
LDL-C (mmol/L)	2.82 (2.33–3.34)	2.87 (2.47–3.40)	0.068	0.318

^*^P < 0.05, ^**^P < 0.01, ^***^P < 0.001.

WHR, waist-to-hip ratio; BMI, body mass index; T2DM, type 2 diabetes mellitus; HTN, hypertension; OSA, obstructive sleep apnea; MetS, metabolic syndrome; AST, aspartate transaminase; ALT, alanine transaminase; UA, uric acid; HbA1c, glycated hemoglobin; HOMA-IR, homeostatic model assessment of insulin resistance; FFA, free fatty acids; TG, triglycerides; TC, total cholesterol; HDL-C, high-density lipoprotein cholesterol; LDL-C, low-density lipoprotein cholesterol.

### 3.2 Post-operative weight loss outcomes

The weight loss outcomes, expressed as %EWL and %TWL at 1, 3, 6, and 12 months post-operatively, are compared in Table 2. Regarding %EWL, the non-MASH group achieved significantly greater weight loss compared to the MASH group at all follow-up time points. Specifically, the median %EWL in the non-MASH group was consistently higher than that in the MASH group at 1 month (*P* < 0.001), 3 months (*P* < 0.001), 6 months (*P* < 0.001), and 12 months (*P* < 0.001). By 12 months post-surgery, the non-MASH group reached a median %EWL of 129.39%, whereas the MASH group achieved 99.68%. In terms of %TWL, the non-MASH group showed significantly higher values during the early post-operative period compared to the MASH group (1 month: *P* = 0.010; 3 months: *P* = 0.007). However, this disparity diminished over time. At 6 months and 12 months post-operatively, the difference in %TWL between the two groups was no longer statistically significant (32.15% vs. 30.34%, *P* = 0.064; and 40.23% vs. 39.95%, *P* = 0.884, respectively).

**Table 2:** Differences in weight loss between non-MASH and MASH groups.

**Outcome**	**Non-MASH**	**MASH**	***Z*/*t***	***P***
Number	63	163		
%EWL (1M)	40.50% (36.09%−49.32%)	32.40% (25.43%−39.71%)	−5.291	< 0.001
%TWL (1M)	13.86% (12.17%−15.65%)	12.93% (11.50%−14.42%)	−2.580	0.010
Number	62	162		
%EWL (3M)	72.80% (63.84%−82.53%)	55.68% (47.18%−68.05%)	−6.360	< 0.001
%TWL (3M)	24.13% ± 3.30%	22.71% ± 3.95%	2.719	0.007
Number	41	123		
%EWL (6M)	96.18% (82.82%−119.95%)	75.96% (63.73%−88.74%)	−5.416	< 0.001
%TWL (6M)	32.15% ± 4.90%	30.34% ± 5.17%	1.883	0.064
Number	26	99		
%EWL (12M)	129.39% (117.20%−139.91%)	99.68% (88.24%−116.63%)	−5.070	< 0.001
%TWL (12M)	40.23% ± 9.44%	39.95% ± 5.46%	−0.146	0.884

%EWL, the percentage of excess weight loss; %TWL, the percentage of total weight loss.

### 3.3 Univariate analysis of factors influencing post-operative weight loss

To identify potential predictors influencing post-operative weight loss, univariate correlation analysis was performed between baseline characteristics and weight loss outcomes (%EWL and %TWL) at 1, 3, 6, and 12 months (Table 3). The analysis revealed that preoperative BMI and the presence of MASH were the most consistent negative predictors of %EWL. Both variables showed a strong, statistically significant negative correlation with %EWL at all post-operative time points (*P* < 0.001). Greater insulin resistance was also negatively correlated with %EWL at all time points. In contrast, gender demonstrated a persistent positive correlation with %EWL, suggesting better excess weight loss outcomes in female patients. For %TWL, the correlating factors differed from those for %EWL and varied over time. Male gender was associated with higher %TWL at 3 and 12 months post-operatively (*P* < 0.05). The association between preoperative BMI and %TWL changed direction over time: a negative correlation was observed at 1 month (*P* < 0.05), which shifted to positive correlations at 6 and 12 months (*P* < 0.05). Similarly, the presence of MASH was negatively correlated with %TWL only in the early post-operative period (1 and 3 months, *P* < 0.01), with no significant association thereafter.

**Table 3:** Univariate analysis of %EWL and %TWL.

**Outcome**	**Variable**	**1 month**	**3 months**	**6 months**	**12 months**
		***r***	***r***	***r***	***r***
%EWL	Gender	0.175^**^	0.199^**^	0.364^**^	0.269^**^
	Age	0.153^*^	0.098	0.098	0.088
	T2DM	0.046	0.064	0.095	0.058
	HTN	0.084	0.133^*^	0.138	0.167
	OSA	0.068	0.081	0.185^*^	0.147
	MetS	0.058	0.105	0.110	0.015
	WHR	−0.160^*^	−0.204^**^	−0.198^*^	−0.032
	BMI	−0.755^***^	−0.749^***^	−0.798^***^	−0.626^***^
	MASH group	−0.353^***^	−0.426^***^	−0.426^***^	−0.455^***^
	AST/ALT	0.023	0.105	0.039	0.067
	UA	−0.115	−0.187^*^	−0.216^**^	−0.237^**^
	TG	0.040	−0.017	−0.083	−0.009
	TC	−0.038	−0.097	−0.027	−0.031
	HDL-C	0.009	0.018	−0.006	0.018
	LDL-C	−0.067	−0.121	−0.017	−0.113
	FFA	−0.147^*^	−0.161^*^	−0.186^*^	−0.189^*^
	HbA1c	−0.059	−0.133^*^	−0.117	−0.054
	HOMA-IR	−0.185^**^	−0.204^**^	−0.260^**^	−0.198^*^
%TWL	Gender	−0.115	−0.135^*^	−0.031	−0.187^*^
	Age	0.089	−0.003	−0.073	−0.034
	T2DM	−0.005	0.027	0.136	0.035
	HTN	−0.062	−0.004	0.008	−0.088
	OSA	−0.046	−0.052	−0.042	−0.164
	MetS	−0.090	−0.034	0.069	−0.081
	WHR	0.080	0.027	0.041	0.173
	BMI	−0.134^*^	0.074	0.171^*^	0.441^**^
	MASH group	−0.172^**^	−0.182^**^	−0.133	0.020
	AST/ALT	−0.030	0.042	−0.098	−0.025
	UA	0.078	−0.030	0.014	0.137
	TG	0.153^*^	0.058	0.008	0.110
	TC	−0.028	−0.044	0.041	0.164
	HDL-C	−0.112	−0.076	−0.178^*^	−0.088
	LDL-C	−0.016	−0.023	0.086	0.097
	FFA	−0.085	−0.069	−0.013	0.015
	HbA1c	−0.019	−0.078	−0.101	0.012
	HOMA-IR	−0.082	−0.022	−0.008	0.146

^*^P < 0.05, ^**^P < 0.01, ^***^P < 0.001.

T2DM, type 2 diabetes mellitus; HTN, hypertension; OSA, obstructive sleep apnea; MetS, metabolic syndrome; WHR, waist-to-hip ratio; BMI, body mass index; AST, aspartate transaminase; ALT, alanine transaminase; UA, uric acid; FFA, free fatty acids; TG, triglycerides; TC, total cholesterol; HDL-C, high-density lipoprotein cholesterol; LDL-C, low-density lipoprotein cholesterol; HbA1c, glycated hemoglobin; HOMA-IR, homeostatic model assessment of insulin resistance; %EWL, the percentage of excess weight loss; %TWL, the percentage of total weight loss.

### 3.4 Multivariate regression analysis of independent predictors

To identify the independent predictors of post-operative weight loss, stepwise multivariate linear regression analyses were performed. The detailed results are presented in Table 4. Preoperative BMI and the presence of MASH were identified as robust, independent negative predictors of %EWL at all time points (*P* < 0.001). Additionally, preoperative HbA1c levels emerged as a negative predictor at 3 and 6 months (*P* < 0.05). Regarding %TWL, the presence of MASH remained an independent negative predictor of %TWL at all post-operative time points. Preoperative BMI showed a divergent impact on %TWL compared to %EWL. While it was not a significant factor in the very early stage, it became a significant positive predictor of %TWL at 3, 6, and 12 months (*P* < 0.05). Regarding metabolic markers, preoperative HbA1c was identified as a negative predictor of both %EWL and %TWL at 3 and 6 months (*P* < 0.05). TG levels were positively associated with %TWL at 1 and 3 months (*P* < 0.05), while the AST/ALT ratio emerged as a negative predictor of %TWL at 1 and 6 months (*P* < 0.05). Notably, HOMA-IR emerged as a significant negative predictor of %TWL exclusively at the 1-month interval (*P* = 0.047).

**Table 4:** Multivariate linear regression analysis of factors influencing post-operative weight loss.

**Outcome**	**Variable**	**B**	**SE**	**β**	***t***	***P***	**Adjusted *R*^2^**
%EWL (1 month)	Constant	0.871	0.044	–	20.167	< 0.001	0.442
	BMI	−0.011	0.001	−0.584	−10.348	< 0.001	
	MASH group	−0.054	0.016	−0.187	−3.317	0.001	
%EWL (3 months)	Constant	1.429	0.072	–	19.881	< 0.001	0.479
	BMI	−0.015	0.001	−0.553	−10.103	< 0.001	
	MASH group	−0.098	0.022	−0.242	−4.374	< 0.001	
	HbA1c	−0.016	0.007	−0.116	−2.217	0.028	
%EWL (6 months)	Constant	1.896	0.090	–	21.208	< 0.001	0.593
	BMI	−0.020	0.002	−0.646	−11.011	< 0.001	
	MASH group	−0.110	0.031	−0.211	3.552	< 0.001	
	HbA1c	−0.019	0.009	−0.120	−2.161	0.032	
%EWL (12 months)	Constant	1.955	0.114	–	17.205		0.424
	BMI	−0.018	0.003	−0.500	−6.193	< 0.001	
	MASH group	−1.181	0.052	−0.281	−3.483	< 0.001	
%TWL (1 month)	Constant	0.148	0.007	–	20.587		0.086
	TG	0.005	0.002	0.201	2.902	0.004	
	MASH group	−0.011	0.004	−0.196	−2.729	0.007	
	AST/ALT	−0.015	0.007	−0.154	−2.215	0.028	
	HOMA-IR	< 0.001	< 0.001	−0.140	−2.003	0.047	
%TWL (3 months)	Constant	0.246	0.027	–	9.055		0.122
	Gender	−0.011	0.006	−0.126	−1.686	0.094	
	MASH group	−0.022	0.006	−0.250	−3.412	< 0.001	
	HbA1c	−0.006	0.002	−0.185	−2.674	0.008	
	BMI	0.001	< 0.001	0.191	2.461	0.015	
	TG	0.005	0.003	0.142	2.010	0.046	
%TWL (6 months)	Constant	0.002	0.001	–	0.354		0.158
	BMI	0.002	0.001	0.354	4.220	< 0.001	
	MASH group	−0.035	0.010	−0.296	−3.451	< 0.001	
	AST/ALT	−0.039	0.016	−0.199	−2.498	0.014	
	HbA1c	−0.006	0.003	−1.161	−2.030	0.044	
%TWL (12 months)	Constant	0.157	0.040	–	3.918		0.293
	BMI	0.007	0.001	0.588	6.646	< 0.001	
	MASH group	−0.048	0.018	−0.234	−2.640	0.010	

### 3.5 Longitudinal analysis of the impact of MASH on post-operative %EWL

To further evaluate the dynamic impact of preoperative MASH on weight loss trajectories, a general linear model for repeated measures was applied to the subset of 125 patients (26 in the non-MASH group and 99 in the MASH group) who completed all four follow-up visits (1, 3, 6, and 12 months). The estimated marginal means for %EWL are presented in Table 5. The analysis revealed significant main effects for both Time [*F*_(3, 121)_ = 486.778, *P* < 0.001] and Group [*F*_(1, 123)_ = 36.086, *P* < 0.001], indicating that %EWL changed significantly over the follow-up period and differed significantly between the MASH and non-MASH groups. More importantly, a significant Time × Group interaction effect was observed [*F*_(3, 121)_ = 11.343, *P* < 0.001]. This suggests that the pattern of weight loss over time was distinct between the two groups. Specifically, the disparity in %EWL between the non-MASH and MASH groups widened progressively from 1 month to 6 months before stabilizing at 12 months.

**Table 5:** Estimated marginal means of %EWL at different time points in MASH and non-MASH groups.

**Time point**	**Group**	***N***	**Mean (%)**	**SE (%)**	**95% CI (%)**
1 Month	Non-MASH	26	44.40	2.43	(39.60, 49.21)
	MASH	99	33.89	1.24	(31.43, 36.35)
3 Months	Non-MASH	26	77.34	3.38	(70.65, 84.03)
	MASH	99	58.25	1.73	(54.82, 61.68)
6 Months	Non-MASH	26	106.22	4.03	(98.25, 114.19)
	MASH	99	77.31	2.06	(73.22, 81.39)
12 Months	Non-MASH	26	129.49	4.58	(120.43, 138.55)
	MASH	99	101.66	2.35	(97.02, 106.30)

SE, standard error; CI, confidence interval.

**Figure 2:**
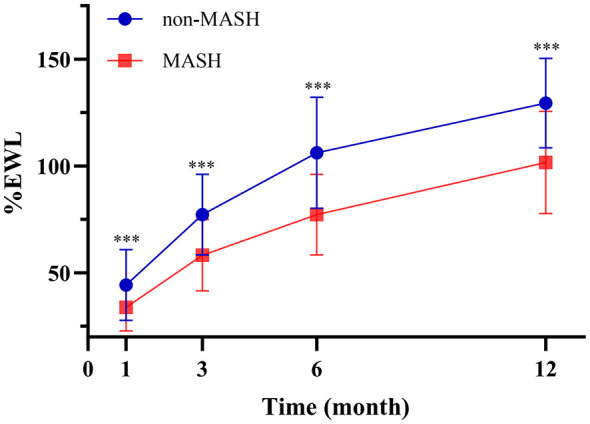
Excess weight loss (%EWL) after sleeve gastrectomy in the two groups.

Simple effect analysis (Table 6) confirmed that the non-MASH group achieved significantly higher %EWL compared to the MASH group at all post-operative time points (*P* < 0.001 for all). The mean difference in %EWL between the two groups was 10.5% at 1 month, increasing to 19.1% at 3 months, 28.9% at 6 months, and remaining substantial at 27.8% at 12 months. To provide a clearer visualization of these trends, the dynamic changes in post-operative weight loss are illustrated in Figure 2. It is noteworthy that, in contrast to the findings for %EWL, the general linear model did not observe a significant main effect of the MASH group on post-operative %TWL [*F*_(1, 123)_ = 0.908, *P* = 0.342].

**Table 6:** Simple effect analysis of %EWL differences between groups.

**Time point**	**Mean difference (%) (non-MASH vs. MASH)**	**SE (%)**	**95% CI (%)**	***P***
1 Month	10.51	2.73	(5.12, 15.91)	< 0.001
3 Months	19.09	3.80	(11.57, 26.61)	< 0.001
6 Months	28.91	4.52	(19.96, 37.87)	< 0.001
12 Months	27.83	5.14	(17.65, 38.01)	< 0.001

SE, standard error; CI, confidence interval.

### 3.6 Kaplan–Meier analysis of time to achieve successful weight loss

**Figure 3:**
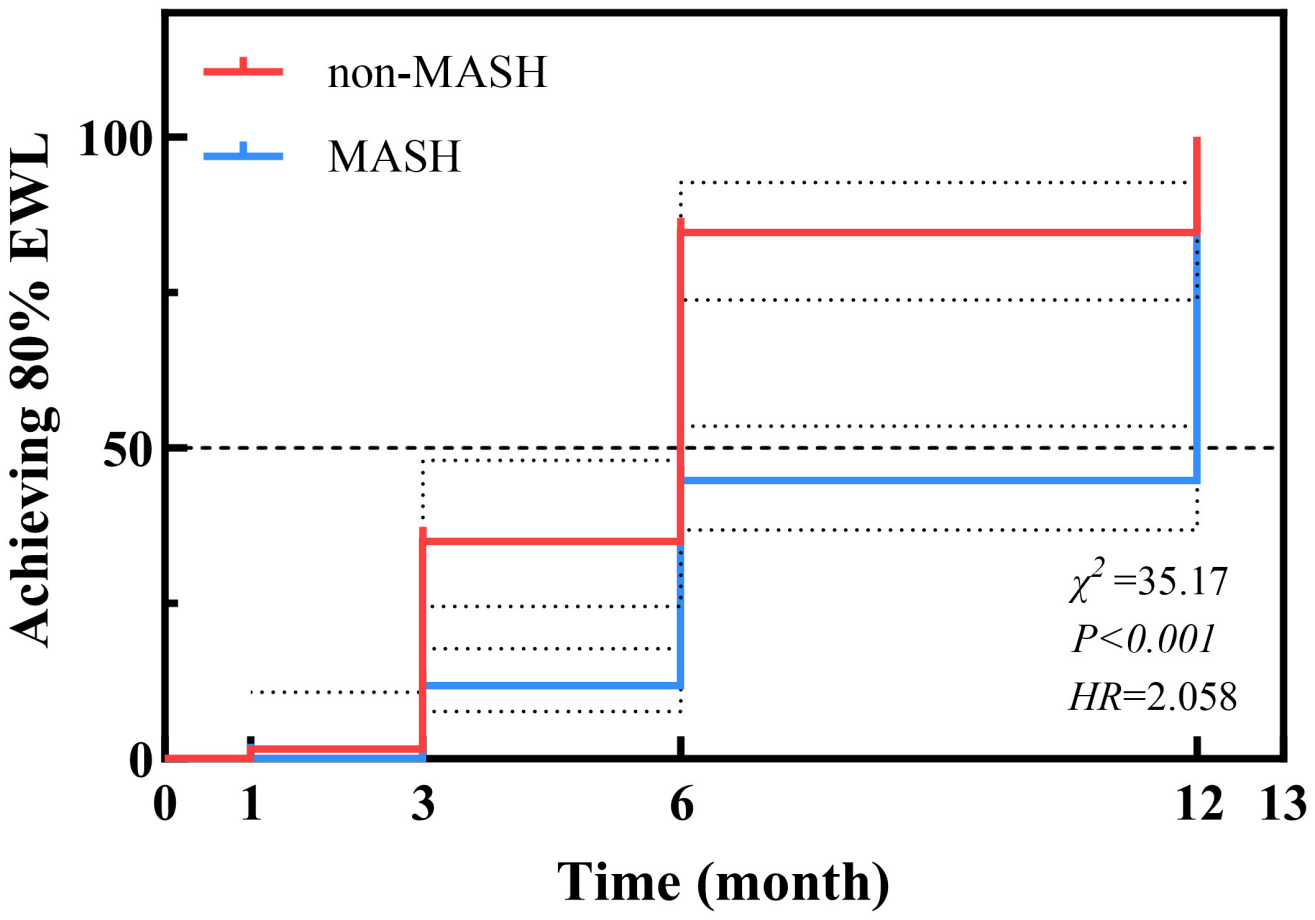
Kaplan–Meier analysis of the cumulative incidence of achieving ≥80% EWL stratified by MASH status.

The time required to achieve a successful weight loss outcome, defined as %EWL ≥80% according to current Chinese guidelines (21), was compared between the groups using Kaplan–Meier analysis. The cumulative incidence of achieving this threshold differed significantly between patients with and without MASH (Log-rank χ^2^ = 35.17, *P* < 0.001; *HR* = 2.058). As illustrated in Figure 3 and Table 7, the cumulative incidence of successful weight loss events was markedly lower in the MASH group compared to the non-MASH group at the same post-operative time points. Specifically, by 6 months post-surgery, 84.7% of patients in the non-MASH group had achieved successful weight loss, whereas only 44.7% of those in the MASH group had reached this milestone.

**Table 7:** Cumulative incidence of achieving 80% EWL at different post-operative time points.

**Group**	**1 Month**	**3 Months**	**6 Months**	**12 Months**
Non-MASH	1.6%	34.9%	84.7%	100%
MASH	0	11.7%	44.7%	84.8%

## 4 Discussion

Our study identifies preoperative MASH as an independent and persistent negative predictor of %EWL within the first year following LSG. Compared to non-MASH patients, those with MASH consistently exhibited significantly lower %EWL at all post-operative time points and required a prolonged period to achieve successful weight loss targets (%EWL ≥ 80%). Notably, this divergence progressively widened during the first 6 months. Although the disparity in %TWL narrowed over time, the deleterious impact of MASH on %EWL remained robust, independent of established confounders such as preoperative BMI and insulin resistance. These findings suggest that the hepatic pathological state itself may directly modulate the efficiency of metabolic benefits derived from bariatric surgery through distinct mechanisms.

Previous studies have consistently demonstrated that bariatric surgery significantly ameliorates MASLD activity and fibrosis risk, and can even promote the remission of NASH/MASH (22, 23). Classical prospective studies and meta-analyses have confirmed that surgery effectively improves steatosis, inflammation, ballooning, and fibrosis, with these improvements closely linked to weight loss and metabolic benefits (22, 24). The current clinical focus has shifted from whether surgery improves liver outcomes to which patients benefit more completely and what kind of perioperative stratified management is required. However, although the beneficial effects of LSG on patients with MASH are well-documented in existing literature, only a few studies have investigated how the presence of MASH influences post-operative weight loss. Based on liver pathology as the gold standard, this study systematically analyzed the influence of preoperative MASH on weight loss outcomes following LSG.

Our multivariate linear regression analysis confirmed that preoperative MASH status and BMI are independent negative predictors of post-operative %EWL and %TWL, indicating a significant correlation between preoperative MASH and reduced weight loss following LSG. Our findings align closely with recent high-quality evidence, particularly the biopsy-based prospective study by Martínez-Montoro et al., which also focused on sleeve gastrectomy (SG) (25). Consistent with their results, we observed that MASH significantly impairs %EWL. By validating these findings in an Asian cohort, our study reinforces the universality of the negative interaction between MASH and bariatric outcomes. Furthermore, our results support the observations of Sabench et al., who suggested that while Roux-en-Y gastric bypass (RYGB) might mitigate the metabolic disadvantages of MASH, SG outcomes remain more susceptible to hepatic inflammatory status (26). In contrast, Abbassi et al., utilizing propensity score matching in RYGB patients, argued that MASH *per se* did not influence outcomes, attributing observed differences solely to metabolic confounders (27). However, within the specific context of LSG, our data challenges this view. Instead, our findings resonate with the large-scale cohort study by Abu-Rumaileh et al., where non-alcoholic fatty liver disease (NAFLD) remained a persistent predictor of inferior weight loss over 5 years, suggesting that intrinsic hepatic mechanisms are indeed at play (28).

Furthermore, preoperative indicators such as HOMA-IR and HbA1c significantly influenced weight loss outcomes at certain time points. This suggests that metabolic dysregulation is not only closely linked to MASH but may also directly interfere with the therapeutic efficacy of bariatric surgery. These findings align with established pathophysiological mechanisms. MASH is not merely an isolated hepatic disease but rather the hepatic manifestation of systemic metabolic dysregulation, characterized by severe insulin resistance, dyslipidemia, and a state of chronic low-grade inflammation. In our cohort, the MASH group exhibited significantly higher levels of WHR, BMI, TG, and HOMA-IR, reflecting a more profound metabolic abnormality and a heavier burden of visceral adiposity. This chronic metabolic and inflammatory environment may impede post-operative weight loss on multiple levels. Previous studies have demonstrated that MASH-associated metabolic abnormalities—including elevated HOMA-IR and lipid disorders—can limit the efficiency of post-operative energy metabolism, thereby hindering weight loss (3, 5, 29). Additionally, the chronic inflammatory state accompanying MASH may further decelerate the restoration of metabolic health after surgery (30).

Figures 1, 2 further illustrate the dynamic interaction between post-operative time and MASH status. The negative impact of MASH on %EWL was most pronounced between 1 and 6 months post-surgery, with the disparity peaking at 6 months. At this time point, the MASH group exhibited an average %EWL that was 28.9% lower than that of the non-MASH group. Although this difference narrowed slightly by 12 months, it remained substantial at 27.8%. These results confirm that the influence of MASH on weight loss outcomes is predominantly concentrated in the early post-operative phase, providing a crucial basis for developing targeted post-operative intervention strategies (26). Furthermore, the model did not observe significant dynamic differences in %TWL based on MASH status. This suggests that the underlying mechanisms governing %EWL and %TWL changes may differ, which is consistent with previous reports. However, the exact mechanisms warrant further investigation (31, 32).

LSG not only promotes weight reduction but also exerts direct regulatory effects on insulin sensitivity and lipid metabolism (33). Our study demonstrates that, although post-operative outcomes were comparatively attenuated in patients with MASH, both %EWL and %TWL still increased significantly over time. This provides compelling evidence that LSG remains an effective therapeutic option for obese patients with comorbid MASH. Furthermore, the potential of bariatric surgery to halt or reverse the progression of MASH has significant implications for long-term liver outcomes. Recent evidence highlights that by inducing substantial weight loss and improving metabolic parameters, procedures like sleeve gastrectomy can effectively downstage liver disease, thereby reducing the future burden of end-stage liver disease. Notably, a predictive modeling study by Rouhi et al. demonstrated that sleeve gastrectomy significantly decreases the projected need for liver transplantation in patients with obesity and MASH (34). This underscores that despite the slightly attenuated weight loss response we observed, the procedure remains critical for mitigating the risk of decompensated cirrhosis in this high-risk population. Nevertheless, to optimize surgical efficacy, attention should be directed toward the preoperative correction of metabolic abnormalities. For instance, preoperative adjuncts such as pharmacological interventions or lifestyle modifications could be employed to ameliorate liver inflammation, insulin resistance, and dyslipidemia (35).

This study has several strengths. We utilized liver biopsy as the gold standard for MASH diagnosis and integrated multi-time-point follow-up data to comprehensively analyze the dynamic impact of preoperative MASH status on LSG efficacy. Consequently, our findings offer significant value for clinical guidance. However, certain limitations should be acknowledged. First, the sample size was relatively limited, particularly in the non-MASH group. Second, the follow-up duration was restricted to 12 months, preventing the assessment of longer-term weight loss and metabolic improvements. Third, due to ethical constraints regarding post-operative invasive procedures, we could not obtain post-operative liver pathology. This precluded the evaluation of histological changes in the liver and limited our ability to elucidate the potential mechanisms underlying MASH improvement. Fourth, to isolate the independent effect of sleeve gastrectomy, we excluded patients who utilized post-operative weight-loss medications or underwent revision surgery. While this strengthens the internal validity of our findings regarding surgical efficacy, it limits the generalizability to real-world scenarios where multimodal therapy (surgery combined with pharmacotherapy) is becoming increasingly common. Future studies should aim to expand sample sizes, extend follow-up periods, and incorporate multi-omics analyses to explore the complex mechanisms linking MASH to bariatric surgical outcomes. Furthermore, investigating specific interventions to optimize preoperative metabolic status will be crucial for improving the prognosis of patients with comorbid MASH.

In summary, our study underscores the significant impact of MASH status on the efficacy of bariatric surgery, particularly during the early post-operative phase. Based on these findings, we advocate for a strengthened, individualized treatment strategy for patients with comorbid MASH. This should encompass a systematic preoperative assessment of liver function, metabolic abnormalities, and insulin resistance, followed by optimized perioperative management through nutritional support, pharmacological intervention, or lifestyle modifications. Furthermore, intensified metabolic monitoring and intervention are crucial during post-operative follow-up, especially within the critical 1-to-6-month window, to maximize surgical benefits.

## 5 Conclusion

This study systematically evaluated the impact of preoperative MASH status on the efficacy of LSG. We found that the presence of MASH was significantly associated with reduced weight loss within the first post-operative year, with the %EWL being 27.8% lower on average compared to non-MASH patients. These findings suggest that MASH status and its associated metabolic abnormalities must be fully considered when assessing the prognosis of obese patients undergoing bariatric surgery. Consequently, individualized treatment and follow-up plans are essential to maximize surgical efficacy. Future research should focus on optimizing preoperative metabolic intervention strategies for patients with MASH to provide a more robust scientific basis for enhancing their weight loss and metabolic outcomes.

## Data Availability

The raw data supporting the conclusions of this article will be made available by the authors, without undue reservation.
